# Novel Biomolecule‐Infused Gelatin Injectable for Treatment of Recurrent Laryngeal Nerve Injury

**DOI:** 10.1002/lary.32459

**Published:** 2025-09-15

**Authors:** Ananya Tadikonda, Sunjay Anekal, Lena W. Chen, Gabriel Sobczak, Troy Wesson, Carmilya Jackson, Marisa A. Egan, Julie C. Liu, Patrick R. Finnegan, Stacey Halum

**Affiliations:** ^1^ Indiana University School of Medicine (IUSM) Indianapolis Indiana USA; ^2^ Department of Otolaryngology‐Head and Neck Surgery IUSM Indianapolis Indiana USA; ^3^ Meharry Medical College Nashville Tennessee USA; ^4^ Davidson School of Chemical Engineering Purdue University West Lafayette Indiana USA; ^5^ Weldon School of Biomedical Engineering Purdue University West Lafayette Indiana USA

**Keywords:** motor end plates, nerve regeneration, UVFP

## Abstract

**Objective:**

Unilateral vocal fold paralysis (UVFP) due to recurrent laryngeal nerve injury (RLN) is a major cause of voice disorders. We have recently identified three biomolecules (agrin, acetylcholine, and neuregulin) with the potential to promote reinnervation after RLN injury. This study aimed to determine if a gelatin injectable with the reinnervating biomolecules will induce site‐specific amplification of neurotrophic factor release and reinnervation after RLN injury in unilateral vocal fold paralysis.

**Methods:**

C57BL/6 mice underwent RLN transection with the following treatment allocations: saline control (*N* = 16), biomolecule cocktail only (*N* = 16), and biomolecule‐infused gelatin (*N* = 16). All injectables were delivered into the denervated thyroarytenoid muscle. Assessment of glottic function was determined via laryngeal electromyography (L‐EMG) and stimulated video laryngoscopy post‐RLN transection on days 7 and 28. Histopathology analysis via immunohistochemistry (IHC) and genetic analysis via quantitative polymerase chain reaction (qPCR) were utilized to characterize reinnervation and gene expression changes within the harvested larynges.

**Results:**

Both biomolecule treatment groups had enhanced reinnervation of the adductor complex, as indicated by higher stimulated L‐EMG area under the curve (*p* < 0.001), adduction during stimulation (*p* < 0.002), midline resting position (*p* < 0.002), and the presence of innervated neuromuscular junctions on IHC. qPCR results suggest both biomolecule treatments resulted in elevated neurotrophic and angiogenic factor expression from the injected muscle.

**Conclusions:**

Injectable biomolecule‐infused gelatin may serve as a novel long‐term therapeutic for glottic functional restoration by redirecting existing mechanisms of synkinesis via site‐specific neurotrophic factor release.

**Level of Evidence:**

N/A.

## Graphical abstract

Biomolecule‐infused gelatin shows promising nerve regeneration, attenuation of muscle atrophy, and functional electromyography outcomes after recurrent laryngeal nerve injury. This biomolecule cocktail may serve as a possible long‐term treatment for vocal fold paralysis and warrants further research in additional animal models.

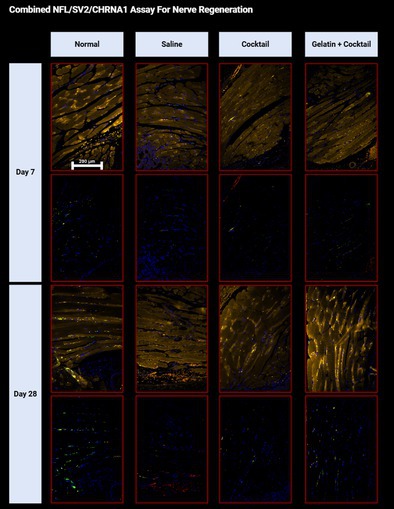

## 1 Introduction

Vocal fold paralysis (VFP) is frequently encountered in otolaryngology practice, negatively impacting patients' quality of life by impairment of phonation and swallowing [1, 2, 3, 4]. Recurrent laryngeal nerve (RLN) damage is one of the most common etiologies of VFP, often resulting from iatrogenic injury, malignancy, and/or trauma [2, 3, 4]. Most surgical interventions for VFP temporarily medialize the paralyzed vocal fold with injectables [5, 6, 7, 8], or include or implantation of synthetic materials through the laryngeal cartilage framework [9, 10]. These interventions do not offer restoration of normal glottic function (motion). Laryngeal reinnervation to restore vocal fold adduction is an alternative intervention; however, it is technically challenging, not offered by many surgeons, and voice outcomes may be subpar due to synkinesis [11, 12]. The limitations of current VFP management warrant the development of a biotherapeutic injectable that medializes the injured vocal fold, promotes laryngeal reinnervation, and minimizes synkinesis to achieve targeted restoration of glottic function.

Recently, the implementation of muscle progenitor cells (MPCs) and their derivatives in animal models of VFP have been explored by our laboratory [13, 14, 15, 16, 17, 18, 19, 20, 21, 22, 23, 24, 25]. MPCs are undifferentiated precursor skeletal muscle cells that include peripheral satellite cells and myoblasts [13, 14, 15]. Motor endplate‐expressing cells (MEEs) consist of differentiated MPCs that release neurotrophic factors necessary for reinnervation [16, 17, 18, 19, 20, 21, 22, 23, 24, 25]. The discovery of MEEs as a therapeutic cell type was initially driven by research on the natural pathophysiology of human neuromuscular junctions (NMJs). Following sudden muscle injury, myofiber endplates lose post‐synaptic signals. In response, the myofibers with denervated endplates release a cascade of physiological neurotrophic factors (NFs) to promote axonal sprouting, neuronal regeneration, and the re‐establishment of functional NMJs [26, 27, 28, 29]. Exogenously administered MEEs are believed to restore laryngeal adductor function after RLN injury through amplified site‐specific NF release (neurotrophic factor‐3 [NTF3], neurotrophic factor‐4 [NTF4], neurturin [NRTN], and nerve growth factor [NGF]) [16, 17, 18, 19, 20, 21, 22, 23, 24, 25].

**FIGURE 1:**
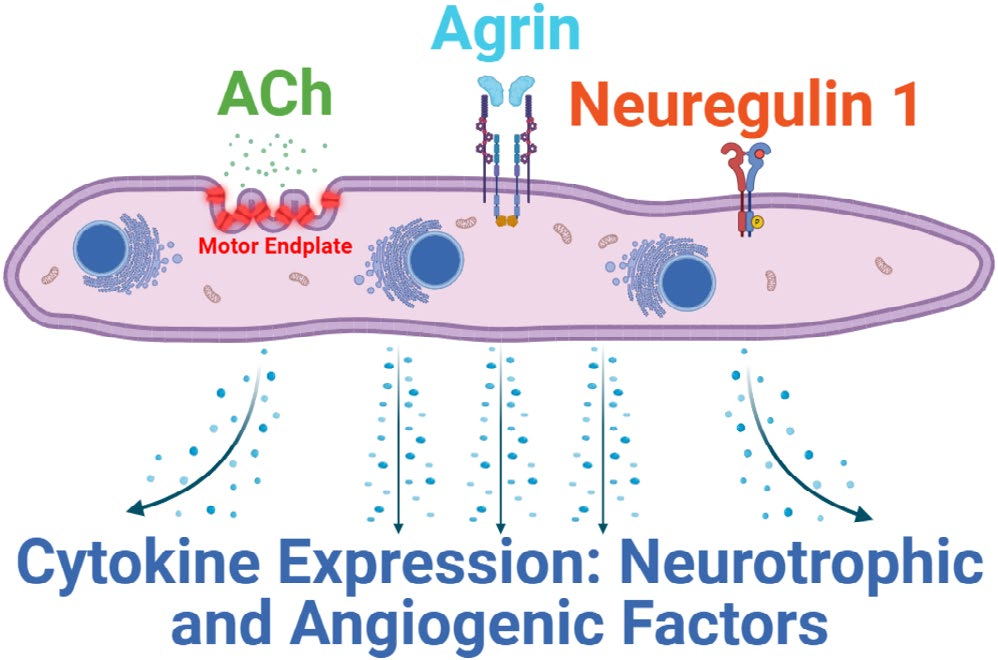
Illustration of the mechanism for agrin, acetylcholine, and neuregulin inducing MPCs to become MEEs with release of neurotrophic and angiogenic factors. Agrin acts through the lipoprotein receptor‐related protein 4 (Lrp4) and muscle‐specific receptor tyrosine kinase (MuSK) to promote clustering and stabilization of acetylcholine receptors (AChRs) [38, 39, 45]. Neuregulin 1 acts via ErbB receptors leading to elevated AChR expression and stabilization of the motor endplate [47, 48, 49, 50]. Finally, with AChRs being activity dependent, the presence of ACh binds the clustering receptors for activation and stabilization [38].

**FIGURE 2:**
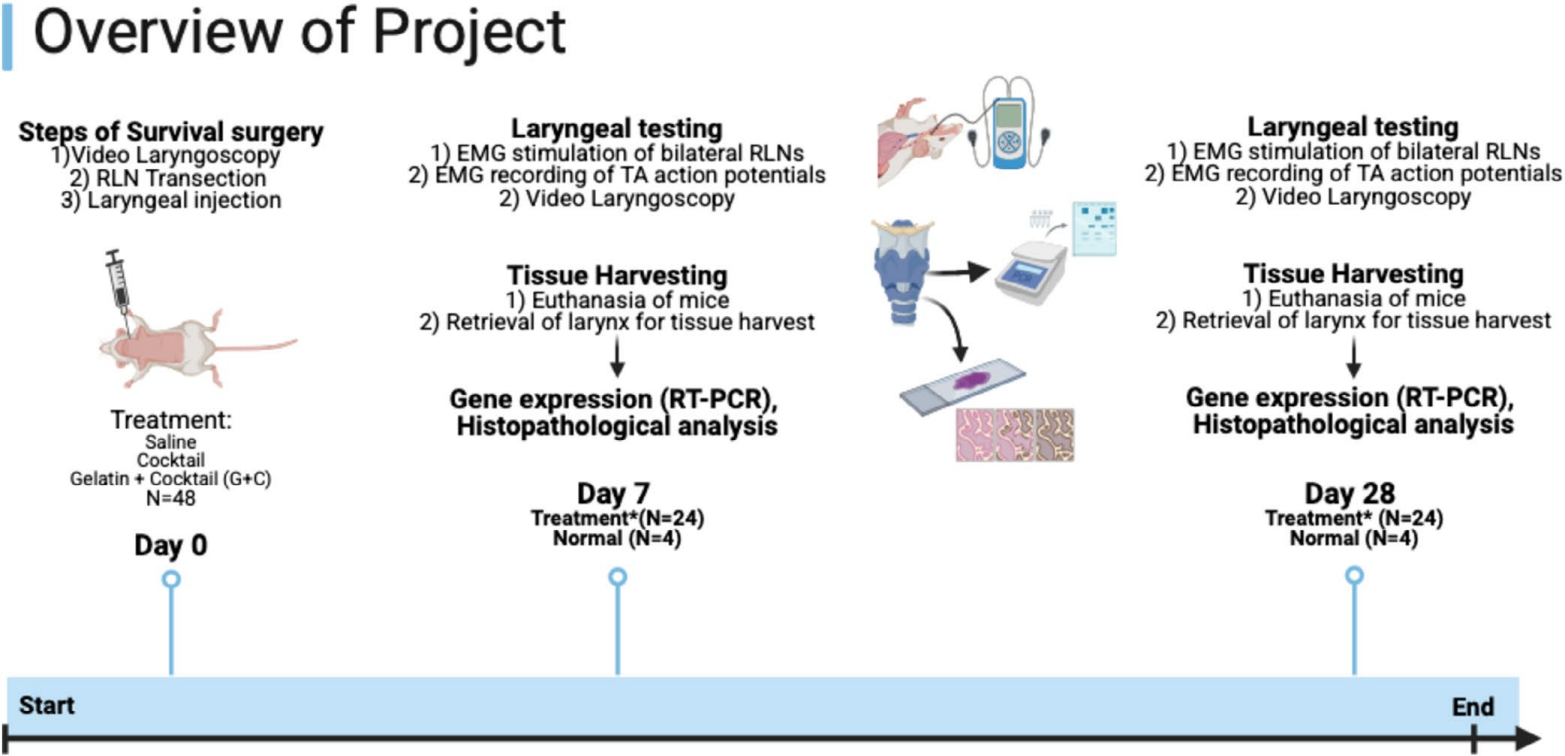
Overview of project indicating the timing of survival surgeries, laryngeal testing, and tissue harvesting on days 0, 7, and 28 after injection of saline, cocktail, and G + C treatment groups. Asterisk refers to the treatment groups outlined under the day 0 description and includes: Saline, cocktail, and G + C. An additional 4 mice were used at 7‐ and 28‐day time points as normal controls that did not undergo transection as shown in image above.

Priming MPCs to differentiate into MEEs through exposure to a biomolecular cocktail of agrin, neuregulin (NRG1), and acetylcholine (ACh) has been thoroughly described [16, 17, 18, 19, 20, 21, 22, 23, 24, 25] and is illustrated in Figure 1. In theory, exogenous injectable administration of these three biomolecules directly into denervated thyroarytenoid (TA) muscle should increase motor endplate expression within the muscle, thereby leading to amplified NF release and selective muscle reinnervation at the injection site. In the current study, we use gelatin as a biomolecule delivery system for delayed site‐specific release of agrin, NRG1, and ACh with the goal of both augmenting and selectively promoting reinnervation of a paralyzed vocal fold in a rodent model. Gelatin has been shown to be an effective delivery system for otolaryngological applications and has demonstrated regenerative properties with minimal inflammatory side effects [30, 31, 32]. This study aims to create a gelatin‐infused injectable containing agrin, NRG1, and ACh, and evaluate its impact on reinnervation and attenuation of atrophy in a rodent model of RLN injury. We hypothesize that injection of the biomolecules (with or without gelatin) into the paralyzed vocal fold will result in: (1) increased motor‐endplate presence on histopathological analysis; (2) increased NF expression on qPCR analysis; (3) enhanced electromyography (EMG)‐based reinnervation compared to controls; and (4) attenuated muscle atrophy in biomolecule treatment groups. We further hypothesize that the gelatin‐biomolecule infusion will promote sustained, site‐specific NF release by delaying biomolecule diffusion at the injection site, compared to the biomolecule cocktail alone. Figure 2 outlines the study timeline along with treatment groups.

## 2 Methods

### 2.1 Injection Preparation

Injections were prepared and loaded for the following treatment groups: 0.9% saline, cocktail, and gelatin + cocktail (G + C). The cocktail solution was prepared with the following concentrations of biomolecules in prepared sterile saline: 500 nM agrin (Catalog# 6624‐AG, R&D, Minneapolis, Minnesota) 1 nM neuregulin (Catalog# 5898‐NR, R&D, Minneapolis, Minnesota), and 100 μM of acetylcholine chloride (Catalog# 159170070, Thermo Scientific, Fair Lawn, New Jersey). This solution was used directly for the cocktail only group, and 0.1 g/1 mL of sterile porcine gelatin (Catalog# 9000‐70‐8, Sigma Aldrich, St. Louis, Missouri) was added to the cocktail for the G + C treatment. To characterize the delayed release of agrin from the G + C injectable, a transwell enzyme‐linked immunosorbent assay (ELISA) was performed. See Supporting Information and Figure S1 for additional information, including methods and results of ELISA.

### 2.2 Survival Surgery

A mouse model was selected for these investigations due to its established translational potential [33, 34]. All surgical procedures followed an IACUC‐approved protocol (#23150; approved 09 February 2024). Day 0 for each mouse was determined based on the date of survival surgery for all groups except the normal groups; day 0 for normal groups was based on the date of arrival after a 72‐h acclimation period. On day 0, C57BL/6 mice from the cocktail group (*N* = 16), saline groups (*N* = 16), and G + C groups (*n* = 16) underwent survival surgery in which the right RLN was transected and vocal fold immobility was confirmed with video laryngoscopy. Injections of 10 μL treatments (saline, cocktail only, G + C) were administered through the thyroid cartilage into the TA muscle. The mice were housed in the Laboratory Animal Resource Center (LARC) facility at Indiana University Indianapolis (IUI) following the LARC facility protocol for animal handling and public health service policy. See Supporting Information section for further detail.

### 2.3 Laryngeal Testing

To test whether successful reinnervation and restoration of muscle function occurred in treatment groups, transoral video laryngoscopy using an Ambu aScope 5 Broncho 2.7/1.2 mm single use flexible endoscope (Catalog# 624001000US, Ambu, Columbia, Maryland) was utilized to visualize vocal fold motion, local inflammation, and positioning of the immobile vocal fold (midline vs. lateral). Observations of video laryngoscopy findings were recorded at day 7 and day 28 and compared to recordings on the day of the survival surgery (day 0).

### 2.4 Laryngeal Electromyography (L‐EMG)

In addition to video laryngoscopy, the mice underwent harvest surgery at their respective 7‐ or 28‐day time point. Intraoperatively, muscle action potentials and bilateral stimulation responses (adduction) were recorded from the bilateral TA muscles while a 5.10 mAMP stimulus was applied to the bilateral RLNs. LEMG stimulation was done on the regenerated fibrous nerve band proximal to, distal to, and at the site of injury. The present study utilized the UltraPro S100 EMG system (Catalog# 828‐060800, Natus, Middleton, Wisconsin). See Supporting Information for more detail.

### 2.5 Histology and Immunohistochemistry

After euthanizing the mice, larynges from each mouse were isolated, stored, and fixed with 10% neutral buffered formalin for 24 h. After fixation, the larynges were placed in HistoGel (Catalog# HG‐4000‐012, Richard‐Allan Scientific, Kalamazoo, Michigan) to maintain tissue structure for processing by the IUI Histology Core. Histology slides were created with 5 μm axial sections of the larynges. Immunohistochemistry (IHC) assay development on selected control tissues and the antibodies used for IHC are listed in Supporting Information. Myofiber diameter was measured for each mouse by utilizing Aperio ImageScope Pathology Slide Viewing Software (Leica, Deer Park, Illinois) to identify diameter approximations of myofibers within scanned H&E images.

### 2.6 qPCR

RNA was isolated from two, 20 μm curls from tissue of each animal, and qPCR analysis was performed to quantify right‐sided gene expression in normal, saline, cocktail, and G + C groups at 7‐ and 28‐day time points. Fold change was calculated from ΔΔCt analysis using *Gapdh* as a baseline to represent relative levels of neurotrophic and angiogenic factor expression in comparison to corresponding 7‐ and 28‐day saline control groups. Further details on the qPCR procedure are outlined in Supporting Information.

### 2.7 Statistical Analysis

For all quantitative comparisons of means between treatment groups, a Kruskal–Wallis test and One‐Way Welch's ANOVA were utilized for non‐parametric and parametric data, respectively. Parametric versus non‐parametric data was determined using the Shapiro–Wilk test.

#### 2.7.1 Functional Outcomes

Mean area under the curve (AUC) data was normalized by dividing the right RLN AUC by the normal left RLN AUC for each mouse to obtain a proportion that was used to analyze relative functionality of the injured nerve. The following treatment groups were compared: all 28‐day groups (normal, saline, cocktail, G + C), all 7‐day groups (normal, saline, cocktail, G + C), and all groups from 7‐ and 28‐day timepoints combined. A Mann–Whitney *U* test was utilized to compare the right‐to‐left AUC proportions between independent 7‐ and 28‐day mice within the same treatment group (e.g., 7‐day saline vs. 28‐day saline).

#### 2.7.2 Video Laryngoscopy

A Wilcoxon signed rank test was utilized to compare nonparametric, paired data for pre‐treatment versus post‐treatment vocal fold motion, inflammatory signs, and immobile vocal fold positioning. A Chi‐square test was used to determine the side of response after 5.10 mAMP EMG stimulation of the bilateral RLNs. Midline versus lateralized positioning of the immobile injured vocal fold was compared between treatment groups through a Chi‐square test with post hoc pairwise comparisons between treatment groups. Differences in recorded myofiber diameters between treatment groups were analyzed through a One‐Way Welch's ANOVA with post hoc Games‐Howell analysis.

#### 2.7.3 qPCR

A One‐Way Welch's ANOVA was performed for parametric data to identify statistically significant differences between treatment groups in their relative expression levels compared to respective saline groups at 7‐ and 28‐day. A Kruskal–Wallis test was performed to analyze 28‐day data for NTF5 given that it is the only group that was nonparametric.

## 3 Results

### 3.1 Video Laryngoscopy

**FIGURE 3:**
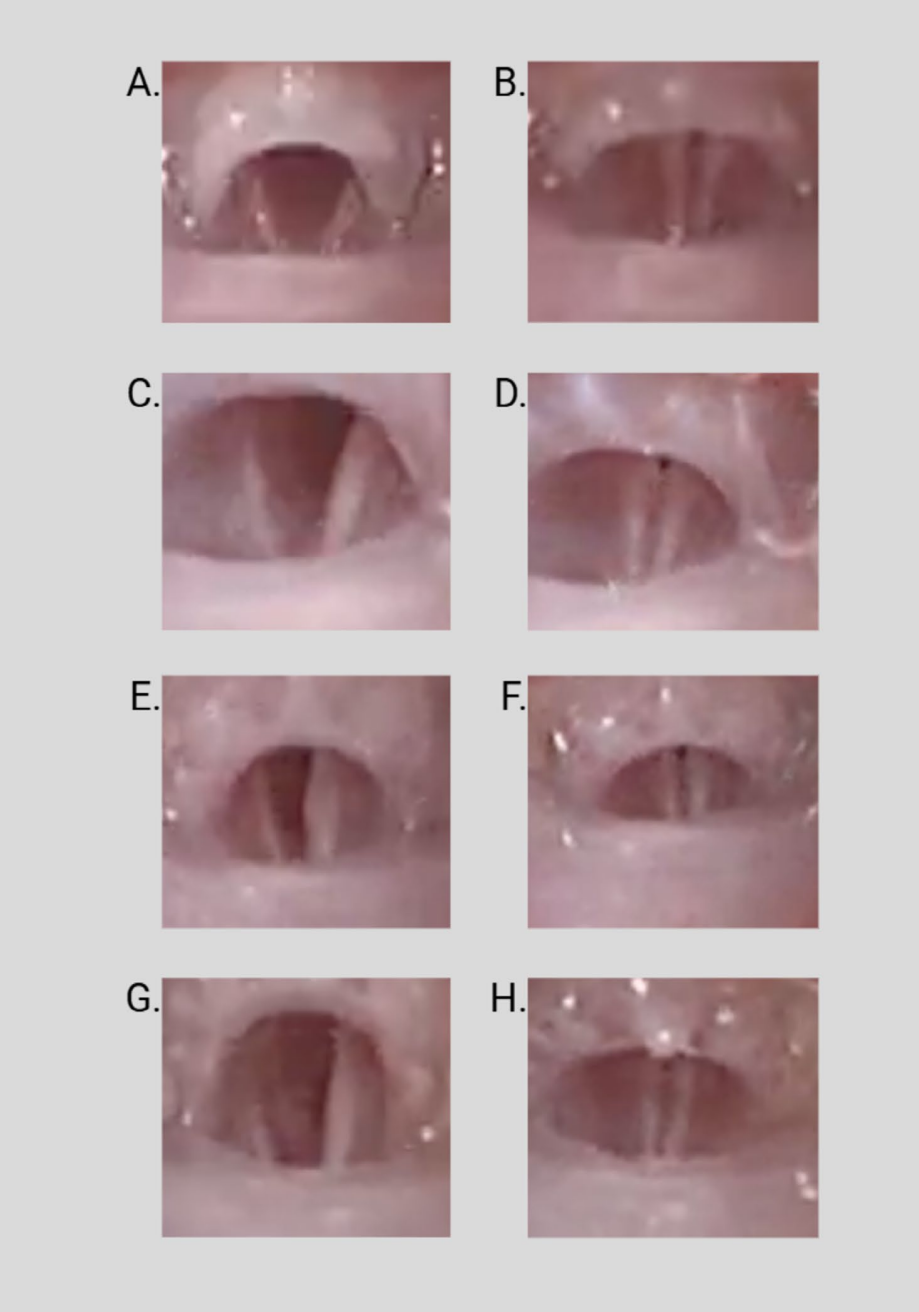
Figure shows video laryngoscopy time points of bilateral vocal folds after 28 days of their respective treatment in each row. Figures are labeled as the following groups: (A, B) normal, non‐transected group, (C + D) saline 28‐day, (E + F) cocktail 28‐day, and (G, H) gelatin + cocktail. The first image of each row displays the vocal fold positioning at full abduction with the second image representing glottic closure. All treatment groups underwent right RLN transection and had an immobile right vocal. The normal mouse did not undergo transection and had a fully mobile right vocal fold.

Blinded analysis of the videos demonstrated the absence of inflammatory changes at 7‐ and 28 days for the cocktail and G + C groups relative to the saline 28‐day group (*p* = 0.408). Bilateral RLN stimulation resulted in a bilateral response (adduction) in all 8/8 cocktail mice and 8/8 G + C groups mice at 28 days in contrast to the saline 28‐day group, which had a response in only 2/7 mice (*p* < 0.001), likely representing functional synkinesis [35]. Specifically, the remaining 5/7 saline 28‐day mice experienced only left unilateral responses with bilateral stimulation, indicating a lack of response from the injured right RLN. All cocktail (7/8) and G + C (8/8) 28‐day mice showed predominantly midline positioning (*p* < 0.002) compared to the saline 28‐day mice, which had mostly lateralized positioning (5/7 mice). Figure 3 displays 28‐day video laryngoscopy images from the normal, saline, cocktail, and G + C groups.

### 3.2 L‐EMG and Nerve Regeneration

**FIGURE 4:**
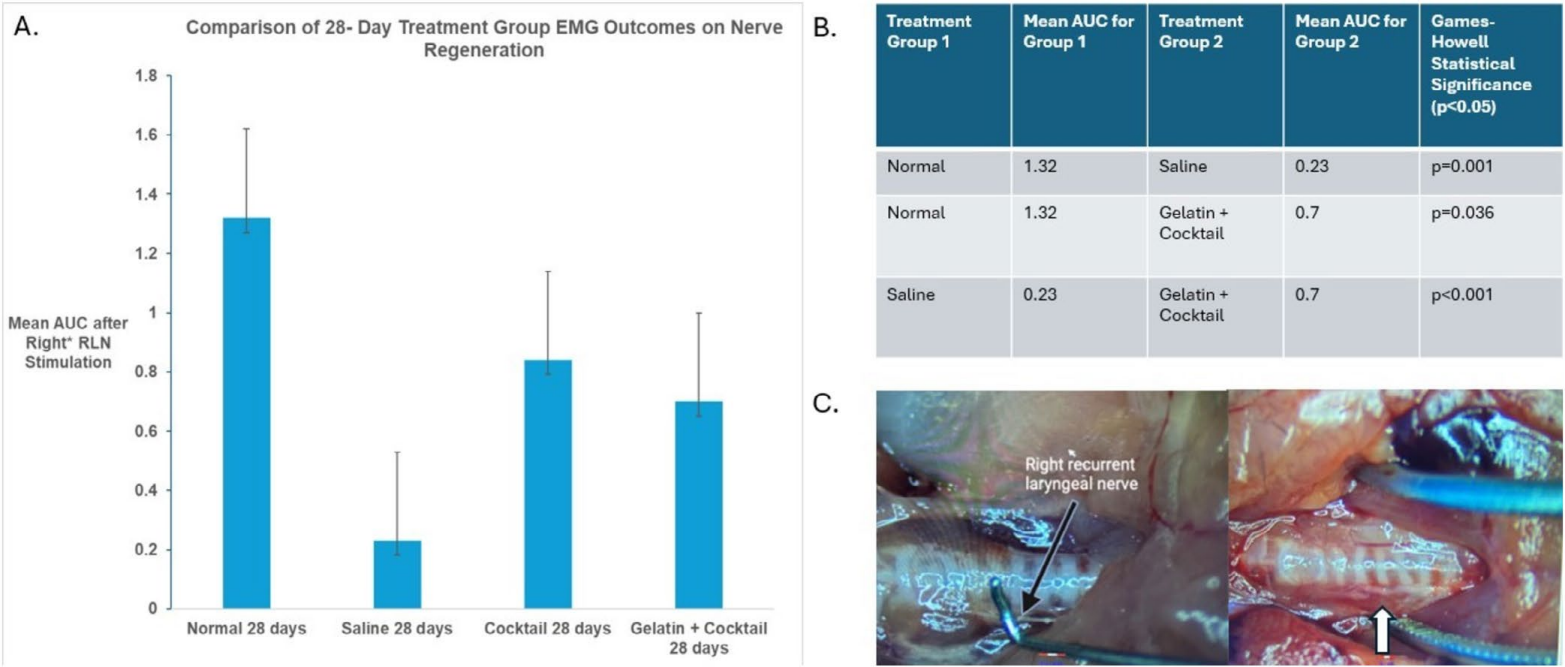
(A) This graph displays the electromyography data after stimulation with 5 mAMP stimulation of the right RLN. The asterisk on graph A is referring to the normalization of the right area‐under‐the‐curve (AUC) to the left for each mouse. (B) Table showing statistically significant differences between the 28‐day treatment groups for the right AUC after 5 mAMP stimulation of right thyroarytenoid muscle. (C) The left image shows isolation of the right RLN prior to transection during the survival surgery compared to the right image of regenerated nerve fibrous band after 28‐day treatment with G + C.

L‐EMG readings were collected at the time of harvest surgery 7‐ or 28‐day after injection with treatment groups. The one‐way ANOVA with post hoc analysis showed a statistical difference between treatment groups at 28 days (*p* < 0.001). Results from this analysis and significant quantitative mean AUC findings from the EMG stimulation across the injured segment of the RLN are listed in Figure 4; the regenerated right RLN fibrous nerve band after 28 days of G + C treatment is also demonstrated. All animals in the 28‐day cocktail and G + C groups had a fibrous band present; however, the G + C bands were thicker and more prominent compared to the cocktail groups.

### 3.3 Histopathological Analysis

#### 3.3.1 Multiplex NFL/SV2/CHRNA1 Assay for Nerve Regeneration

**FIGURE 5:**
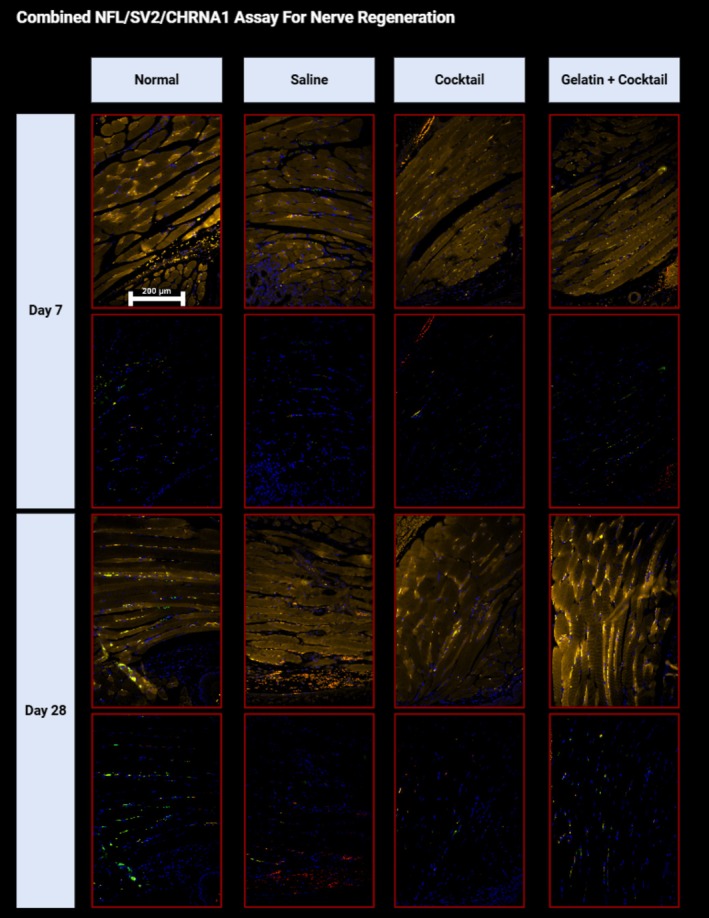
Image displays NFL (455 nm, green)/SV2 (555 nm, red)/CHRNA1 (647 nm, yellow) combined immunofluorescent staining at 400× on the Leica DM2500 microscope to represent nerve regeneration after respective treatments at 7‐ or 28‐day. DAPI (blue) counterstain was used to indicate myofiber nuclei. For each time point, the first row represents the combined NFL/SV2/CHRNA1 assay to show the presence of new NMJs within the selected myofibers. The second row displays only NFL/SV2 to highlight nerve markers signifying nerve presence.

**FIGURE 6:**
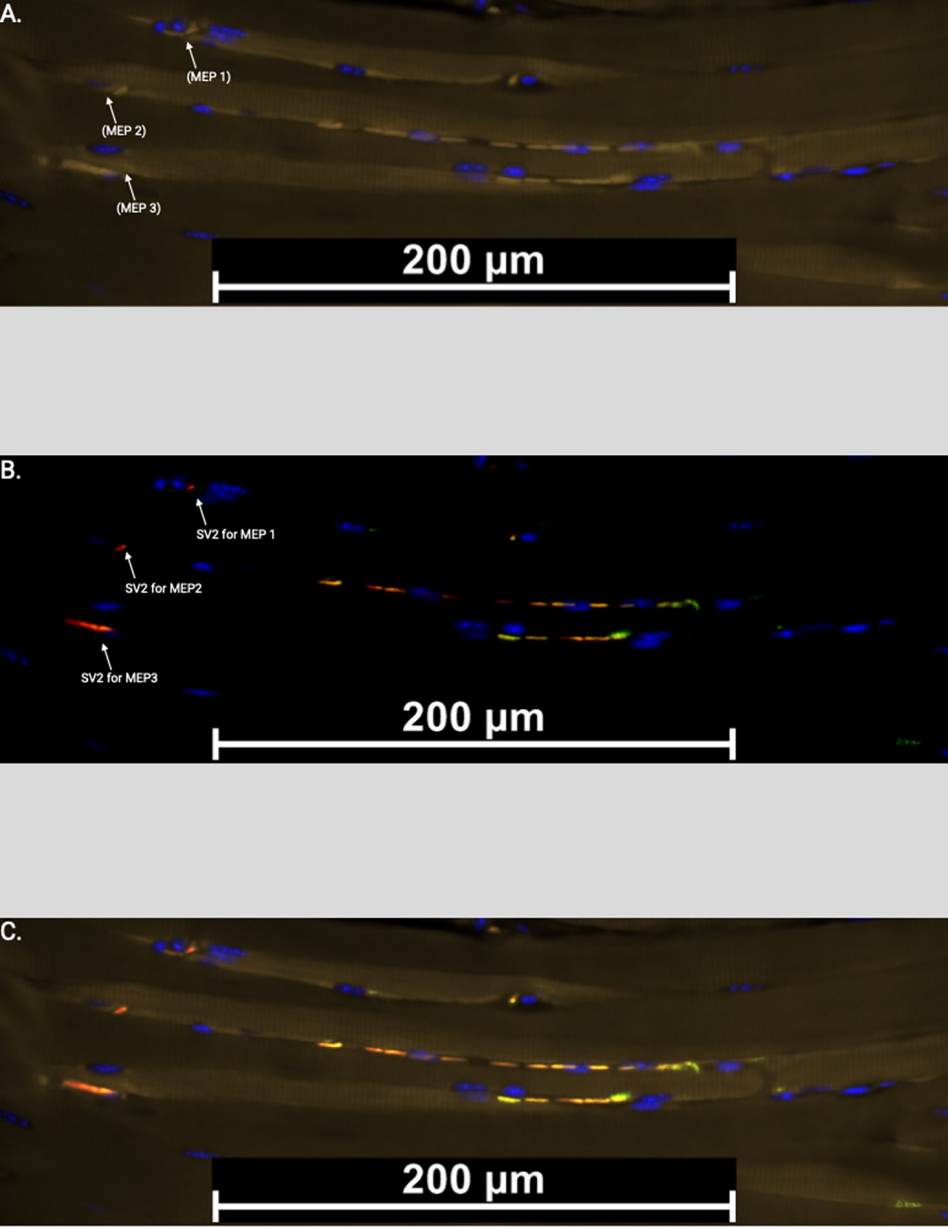
Figure shows combined NFL/SV2/CHRNA1 staining from a G + C, 28‐day mouse at 200× magnification to highlight individual motor end plates (MEPs) at NMJs. Image A is CHRNA1 only with the DAPI counterstain to highlight the presence of MEP 1, MEP 2, and MEP 3 on the respective myofibers. Image B highlights SV2/NFL only with the DAPI counterstain to highlight neuronal regeneration. SV2 staining represents pre‐synaptic vesicles and can be seen adjacent to their associated MEPs from Image A. NFL indicates neuronal presence overall. Image C is the combined NFL/SV2/CHRNA1 stain with all components of the NMJ visible.

Treatment with cocktail and G + C at 7 and 28 days showed a notable increase in innervated NMJs in the denervated muscle, with findings similar to the contralateral (uninjured) side (Figure 5). Not only was diffuse CHRNA1 immunopositivity observed (demarcating motor endplates), but immunopositivity for SV2 was present adjacent to increased CHRNA1 in the myofibers, consistent with innervated NMJs throughout, as shown in Figure 6. NFL, a neuronal marker, was present throughout the muscle with adjacent increases in CHRNA1 and SV2 indicative of nerve and NMJ regeneration at injury site‐specific sites. In contrast, the saline 7‐ and 28‐day groups showed much less SV2 and NFL (neuronal presence) when compared to the normal non‐transected mice (Figure 5).

#### 3.3.2 Multiplex Desmin/MuRF1 Assay for Muscle Integrity and Atrophy

**FIGURE 7:**
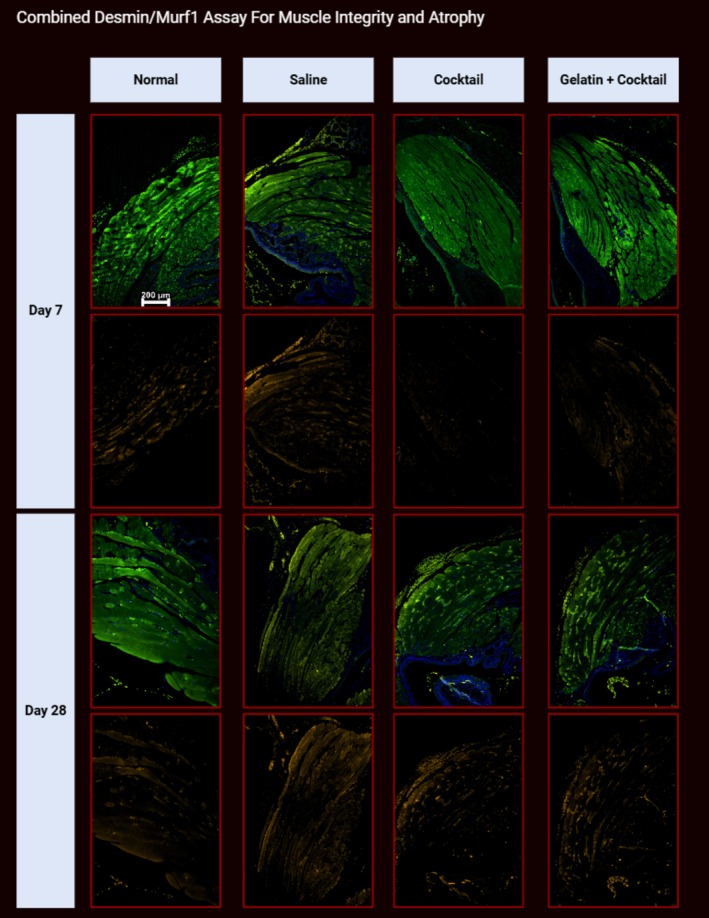
Image displays Desmin (488 nm, green)/Murf1 (555 nm, orange) combined immunofluorescent staining at 100× to represent muscle integrity after 7‐ or 28‐day with their respective treatments. DAPI (blue) counterstain was used to indicate myofiber nuclei. For each time point, the first row represents the Desmin only assay to confirm presence the thyroarytenoid muscle. The second‐row displays Murf1, a measure of muscle atrophy [51], to highlight muscle atrophy in the respective muscle.

**FIGURE 8:**
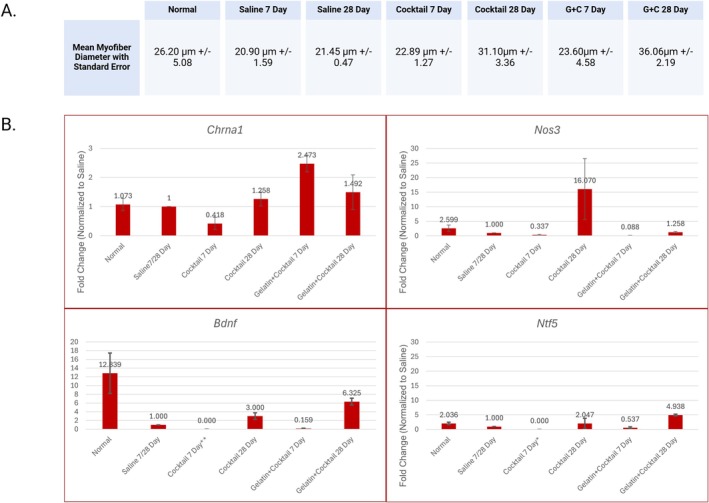
Image A displays a table with mean myofiber diameters for each treatment group with standard errors. Image B displays qPCR fold change for each treatment group for *Nos3*, *Ntf5*, *Chrna1*, and *Bdnf* to represent RNA expression of vascular and nerve regenerative genes. All 7‐day fold change values were compared to the saline 7‐day fold change and all 28‐day time points were compared to the saline 28‐day fold change. The low *Chrna1* levels in the cocktail 7‐day group followed by an increase in *Chrna1* at 28 days may have occurred due an initial high‐dose inhibitory transcriptional effect, which resolved over time as the biomolecule concentration declined into therapeutic values. The gelatin + cocktail groups likely facilitated lower but more consistent biomolecule release over time, maintaining elevated *Chrna1* expression. *Refers to no amplification. **One technical replicate within sample did not amplify.

Saline at 7 days showed minimal muscle atrophy compared to the saline 28‐day groups, which displayed significant atrophy and muscle damage. Specifically, saline 28‐day groups showed a significant increase in atrophy based on MuRF‐1 immunopositivity on the injured side compared to the contralateral uninjured muscle. Denervated sides treated with cocktail and G + C showed notably less atrophy at 7‐ and 28‐day time points compared to the saline controls and demonstrated similar muscle morphology as the uninjured side, with similar levels of MuRF1 immunopositivity. Figure 7 shows the comparison of the Desmin/Murf1 staining between treatment groups at 7‐ and 28‐day time points. Myofiber diameter was greater in G + C compared to saline groups at 28 days (mean G + C 36.06 μm ± 2.19 vs. saline 21.45 ± 0.47, *p* = 0.024). Additionally, the G + C 28‐day group had higher myofiber diameters than the saline 7‐day group mean of 20.9 ± 1.59 (*p* = 0.006). Of note, the cocktail 28‐day group had a mean myofiber diameter of 32.1 μm but did not yield statistically significant results. Figure 8 displays a table with all the mean myofiber diameters for each treatment group with standard error values.

### 3.4 Gene Expression Analysis (qPCR)

qPCR analysis is displayed in Figure 8.

#### 3.4.1 Chrna1

Analysis showed both cocktail groups (7‐ and 28‐day) and G + C 28‐day were not significantly changed from the saline control. G + C 7 days had a higher fold change at 2.47 compared to saline. There were no significant differences between the 28‐day cocktail and G + C, though their mean fold changes were 1.258 and 1.492, respectively.

#### 3.4.2 Bdnf

Analysis of 7‐day data showed a lower mean fold change of 0.159 in the G + C group (*p* = 0.010) and near zero in the cocktail 7‐day compared to the saline. The cocktail group at 28‐day had a notable mean fold change of 3; however, it had a *p* value of 0.305. Comparing 28‐day data of *Bdnf* expression of G + C to saline, the G + C showed a significantly higher fold change of 6.33 (*p* = 0.040).

#### 3.4.3 Ntf5

The G + C 28‐day group had a significantly higher fold change of 4.94 in *Ntf5* expression compared to the saline 28‐day group (*p* = 0.016). Cocktail 28‐day and G + C 28‐day groups had fold changes of 2.047 ± 1.75 and 4.938 ± 0.32, respectively; though no statistical differences were noted between these treatments.

#### 3.4.4 Nos3

There was no significant difference noted in (7‐ and 28‐day) treatment groups when comparing *Nos3* fold changes; however, the cocktail 28‐day group had a fold change of 16.07 with a standard error of 10.49.

## 4 Discussion

This study aimed to examine macroscopic, microscopic, and functional outcomes of injecting denervated larynges with biomolecule‐infused gelatin in C57BL/6 mice. Evaluation of the findings shows that injecting acutely denervated laryngeal muscle with agrin, NRG1, and ACh (with or without gelatin) promotes an increase in nerve regeneration, innervated NMJs, and overall muscle integrity.

### 4.1 Functional Outcomes

All injected mice maintained a steady weight, appetite, respiratory status, and no signs of adverse reactions to any injectables. Video laryngoscopy recordings after 7‐ and 28‐day treatments exhibited no significant changes in vocal fold movement in all treatment groups, which reflects an expected outcome at this early timepoint. Specifically, video laryngoscopy findings in Figure 3 and statistical differences of the injured, immobile vocal fold positioning between the negative control saline group and G + C group support synkinesis mechanisms. Lateralized vocal fold positioning of the negative control saline group can be explained by disorganized reinnervation of abductor and adductor muscles possibly leading to a slightly abducted position. In contrast, the midline positioning in the G + C group may be explained by targeted reinnervation mechanisms from the biomolecules leading to stronger innervation of the adductor muscles innervated by the RLN [35, 36, 37].

### 4.2 Biomolecules Promote Reinnervation and Attenuate Atrophy

L‐EMG data demonstrated a significant increase in right AUC of the denervated TA muscle in the G + C group compared to the saline negative control groups after 28 days, signifying regeneration of the injured RLN. Findings are supported by the presence of a fibrous nerve band that led to successful EMG readings, as seen in Figure 4. Bilateral adduction upon stimulation seen in many G + C and cocktail injected animals at 28 days also suggests stimulable innervation had formed in those groups, likely because the regenerated nerve allowed for a stronger site‐specific adductor muscle reinnervation and response.

Immunofluorescent staining examining nerve regeneration and muscle atrophy attenuation showed promising regenerative effects of the cocktail and G + C groups. Immunopositivity for SV2 and NFL was observed adjacent to Chrna1, indicating innervated NMJs throughout the treated muscle, specifically at Chrna1‐upregulated sites. Figure 6 displays how the biomolecules not only contributed to the formation of new innervated MEPs but also increased SV2 markers indicative of regenerated nerve at sites of significant NFL staining of nerve damage. Other areas demonstrated Chrna1 without SV2 and NFL, suggesting non‐innervated motor endplate‐expressing regions. Based on our prior MEE injection studies, these motor endplate‐expressing regions should result in site‐specific neurotrophic factor release [16, 17, 19]. In addition, attenuation of muscle atrophy occurred in both the cocktail and G + C groups, in which increased NMJ formation was evident, promoted muscle integrity, and attenuated muscle atrophy [38, 39].

Biomolecules promote gene expression of *Nos3*, *Chrna1*, *Bdnf*, and *Ntf5*. Comparisons of fold change for QPCR data show significant increases in *Bdnf*, *Chrna1*, and *Ntf5* starting as early as 7 days, with persistent levels of expression at 28 days. Interestingly, the G + C groups resulted in the most consistent, statistically significant increases in fold changes compared to the cocktail groups for *Bdnf*, *Chrna1*, and *Ntf5* expression at 7‐ and 28‐day time points. This data suggests that gelatin as a delivery system of the cocktail may be allowing for sustained release of the biomolecules over a longer period, resulting in increased expression of neurotrophic and angiogenic factors.

Important limitations of this study include genetic differences between mice and humans, the translational potential of the mouse model, and early analysis timepoints. Additionally, gelatin alone has been shown to have anti‐inflammatory and regenerative properties, so adding a gelatin alone control would have strengthened the design [40]. Despite these shortcomings, the current findings suggest additional investigation with variable time between injury and injection, longer follow‐up, and alternative preclinical models is warranted.

Given the microscopic and macroscopic results of this study, it is important to consider each biomolecules' potential mechanism of action. Agrin, NRG‐1, and ACh have each shown promising nerve regeneration and muscle restoration properties through the Agrin‐Lrp4‐MuSK signaling pathway, as outlined in Figure 1. Not only does each biomolecule contribute to synaptogenesis, neurogenesis, and muscle regeneration, but there are synergistic effects between agrin, NRG‐1, and ACh that further reinforce their individual therapeutic benefits [41, 42, 43, 44, 45, 46, 47, 48, 49, 50]. To our knowledge, this investigation is the first to use these three biomolecules together as a therapeutic injectable for laryngeal denervation injury (see Supporting Information).

## 5 Conclusions

The biomolecules agrin, NRG1, and Ach within a gelatin scaffold show promising early outcomes as an injectable treatment for RLN injury. Evidence of nerve regeneration, attenuated muscle atrophy, and functional EMG outcomes outline the robust regenerative capacity of the cocktail. Given the significant response at only 28 days following treatment, further timepoints and applications to other animal models are warranted.

## Ethics Statement

This study was performed in accordance with the PHS Policy on Humane Care and Use of Laboratory Animals, the NIH Guide for the Care and Use of Laboratory Animals, and the Animal Welfare Act (7 U.S.C. et seq.); the Indiana University Institutional Animal Care and Use Committee (IACUC) approved the animal use protocol (23150; Approved 09 February 2024).

## Conflicts of Interest

Dr. Stacey Halum founded Vibrant LLC, a startup that has licensed primed muscle cell manufacturing technology from the Purdue Research Foundation for further development and commercialization.

## Supporting information


**Figure S1:** Figure shows setup of transwell plate with 1 mL of gelatin + PBS or gelatin + cocktail mixture in the top well and 3 mL of DMEM in the bottom well. Collagenase was added to the DMEM allowing for breakdown of gelatin and diffusion of biomolecules through the permeable membrane into media.

**Data S1:** Expanded methods, results, and discussion.
