# Supplementary figures and images for: Novel Biomolecule‐Infused Gelatin Injectable for Treatment of Recurrent Laryngeal Nerve Injury
Source: Laryngoscope. 2025 Sep 15;135(12):4781–92. doi: 10.1002/lary.32459 (PMC12706572; doi:10.1002/lary.32459)

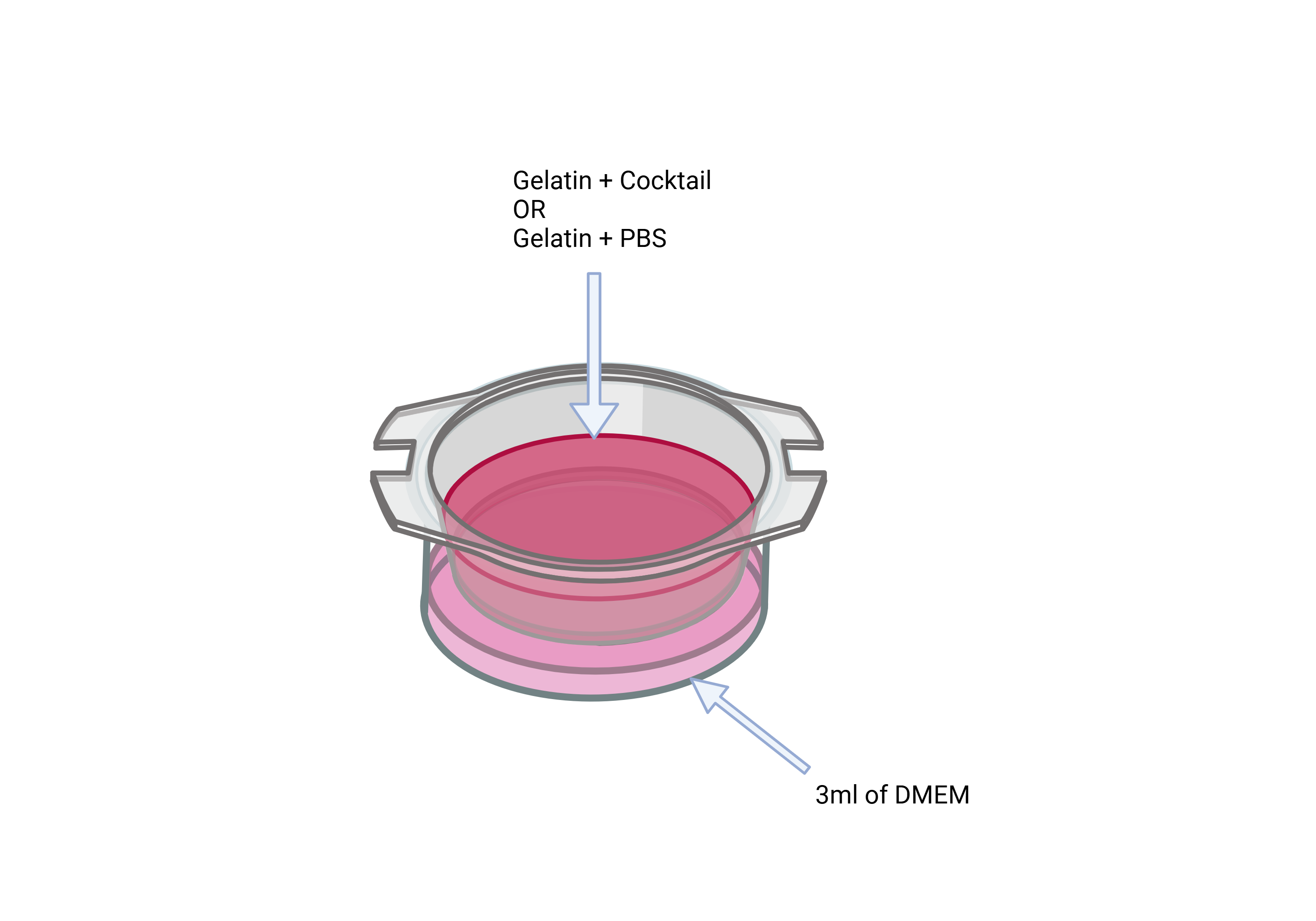

Supplement: Supplementary file 1 — Figure S1: Figure shows setup of transwell plate with 1 mL of gelatin + PBS or gelatin + cocktail mixture in the top well and 3 mL of DMEM in the bottom well. Collagenase was added to the DMEM allowing for breakdown of gelatin and diffusion of biomolecules through the permeable membrane into media. [file LARY-135-4781-s002.png]
