# Supplementary material for: Novel Biomolecule‐Infused Gelatin Injectable for Treatment of Recurrent Laryngeal Nerve Injury
Source: Laryngoscope. 2025 Sep 15;135(12):4781–92. doi: 10.1002/lary.32459 (PMC12706572; doi:10.1002/lary.32459)
Supplement: Supplementary file 2 — Data S1: Expanded methods, results, and discussion. [file LARY-135-4781-s001.docx]

**Supplemental Information**

**Methods**

Assay Development Information:

After utilizing The Human Protein Atlas to identify positive and negative control tissues for each antibody, control human slides were obtained and stained according to a lab-specific IHC protocol that utilized the Novolink Polymer Detection System with 5 minutes of peroxidase block, 30 minutes of antibody binding, 30 minutes of HRP polymer binding, 5 minutes of DAB staining, with a final step of 5 minutes of hematoxylin staining. After determining an ideal antibody concentration based on control slides, the antibodies were also developed on control nude mouse tissue and normal C57 mouse tissue. The slides were analyzed using a Leica DM2500 microscope to detect appropriate immunopositivity or negativity at ideal antibody concentrations based on the quality of staining at different concentrations.

IHC Antibody Information:

The following direct conjugate antibodies were used: anti-SV2A antibody (Catalog#317770, Abcam, Waltham, Massachusetts) to identify neuronal synaptic vesicles after reinnervation; neurofilament (NFL) antibody (Catalog# C28E10, CST, Danvers, Massachusetts) to identify neuronal presence; anti-MURF1 (Catalog# 201941, Abcam, Waltham, Massachusetts) to demarcate areas of muscle atrophy; Desmin antibody (Catalog# 73348S, CST, Danvers, Massachusetts) to confirm the presence of muscle cells; and CHRNA1 (Catalog# 308307, Abcam, Waltham, Massachusetts) to depict acetylcholine receptors within the motor endplates.

Agrin Transwell Study ELISA Data:

Given the importance of agrin within the biomolecule treatment cocktail, an in vitro diffusion study was performed to analyze relative diffusion concentrations of agrin from homogenized gelatin over a 19-day period. The diffusion of agrin was measured within the following treatment groups: Gelatin+PBS and Gelatin+Cocktail. The cocktail consisted of 1 nM recombinant rat agrin (Catalog# 550-AG/CF, R&D, Minneapolis, Minnesota), 2 pM NRG-1 (Catalog# 5898-NR, R&D, Minneapolis, Minnesota), and 1nM acetylcholine (Catalog# 159170070, Thermo Fischer Scientific, Fair Lawn, New Jersey). Transwell plates with 0.4 µm polycarbonate membrane (Catalog# 3412, Costar, Kennebunk, Maine) were utilized as shown in **Supplemental Figure 1.** For days 0-10 no collagenase was administered to the transwell, whereas for days 11-19, 1 mL of collagenase was administered on Day 10 to facilitate gelatin breakdown, mimicking physiological conditions. Around 1-1.5 mL of DMEM containing diffused agrin from gelatin was collected at respective time points from the bottom well. Collected samples were stored at -20°C to be evaluated with an ELISA. After all samples were collected, they were evaluated with a rat agrin ELISA kit (Catalog# ERA2RB, Thermo Fischer Scientific, Carlsbad, California).

QPCR Methods:

Two 20 µm curls were obtained and isolated using the RNeasy FFPE Mini kit (Catalog# 73504, Qiagen, Germantown, Maryland) from laryngeal tissue of mice from each treatment group. RT PCR was performed using the First Strand cDNA Synthesis kit (Catalog# NP100042; Origene Technologies, Inc.; Rockville, Maryland). qPCR was performed using Origene Universal SYBR Green qPCR Master Mix (Catalog# NP100055) and Origene qSTAR Primer Pairs for Chrna1 (Catalog# 202479), Bdnf (Catalog# 201391), Ntf3 (Catalog# 208866), and Nos3 (Catalog# 208934) with Gapdh (Catalog# 205604) used as housekeeping reference gene for ΔΔCt analysis.

Cocktail 7-day and saline 7-day groups had RNA pooled from n=3 mice into one sample whereas the remaining treatment groups had RNA pooled from n=4 mice for collecting *Bdnf*, *Nos3* and *Ntf5* data. Technical replicates for these samples were run in triplicate on the Applied Biosystems ViiA7 Realtime Thermal Cycler to achieve an n=3 for statistical significance. All treatment groups underwent individual isolations with non-pooled RNA for detecting *Chrna1*. Relative expression levels of *Bdnf*, *Nos3*, *Chrna1*, and *Ntf5* were determined from qPCR analysis by acquiring mean cycle threshold (CT) values.

Injection Preparation for Survival Surgeries:

Sterile saline was prepared by measuring out 9 g salt per 1000 mL water. Prepared solution was autoclaved for sterility. Gelatin was added to the cocktail solution on the day of surgery to minimize solidification of the injectate with cocktail injections being stored in 4 C. Injections were loaded at 10 µL in 31-gauge 0.3 mL insulin syringes (NDC# 08290-3284-40, BD, Franklin Lakes, New Jersey).

Survival Surgery Procedure:

During the survival surgery, the mice were initially anesthetized with inhalation of isoflurane and placed on the surgery platform under a Steindorff Digital Video microscope (New York Microscope Company, Hickman, New York).

The ventral neck fur was trimmed with an electric razor. Sterilization of the surgical site was achieved through application of iodine, isopropyl alcohol, and draping with sterile Press’n Seal saran wrap per the IACUC protocol. Transoral video laryngoscopy was performed to confirm normal movement of the vocal folds pre-operatively and served as a reference for post-operative transection confirmation. A vertical, midline incision extending from the sternal notch to the hyoid bone was made on the ventral neck with a #15 blade scalpel. The submandibular gland was exposed and carefully separated using blunt dissection. The infrahyoid muscles were then encountered and carefully separated by sharp dissection and lateralized to expose the pre-tracheal fascia. The fascial layers were delicately separated from the trachea at the level of the fourth through sixth tracheal rings with dental microhooks.

The right RLN was carefully separated from the inferior thyroid artery and transected with micro-scissors. Injections of 10 µl treatments (saline, cocktail only, G+C) were administered to the respective mice through the thyroid cartilage into the thyroarytenoid (TA) muscle. No RLN injury or injection was performed on the normal control mice. The infrahyoid muscles were re-approximated with 6-0 vicryl suture and the midline skin incision was closed with 7mm wound clips. A video laryngoscope was utilized after completion of surgery to verify RLN transection by visualizing the lack of mobility of the ipsilateral vocal fold.

The mice were housed in LARC facility (Laboratory Animal Resource Center) at Indiana University of Indianapolis following the LARC facility protocol for animal handling and public health service policy. Wound checks were performed daily for one week for signs of infection. All animals were additionally monitored daily for signs of pain and/or distress and administered an additional Ethiqa 3.25 mg/kg for analgesia. All animals were given ample housing space with wet food and safe living conditions in accordance with the Animal Welfare Act. Three mice died due to intraoperative or perioperative complications.

L-EMG procedure during Harvest Surgery:

The following stimulation responses were recorded with simultaneous video laryngoscopy during the harvest surgery: right RLN stimulation, left RLN stimulation, and bilateral RLN stimulation. After visualizing adduction responses from stimulation, a 31-guage recording needle electrode was placed through the thyroid cartilage into the right and left TA muscles during stimulation of the ipsilateral nerve to record area-under-the-curve (AUC) values representative of action potential responses. Comparisons of AUC after right RLN stimulation from the EMG recordings were noted at 7 and 28 days to determine the effects of the saline, G+C, and cocktail groups on injured RLN reinnervation and ipsilateral restoration of TA muscle function after RLN injury. After laryngeal testing, the mice were then euthanized with inhaled carbon dioxide gas followed by cervical dislocation.

**Results**

Agrin ELISA showed that after addition of collagenase on Day 10, there was a surge of percent agrin diffusion starting at about 3% on day 13 to 44% diffusion at 19 days. There was no other identifiable diffusion pattern for the first 10 days without collagenase displaying gelatin’s ability to contain the cocktail in a homogenized form. Surge in diffusion after 13 days displays that the drug can be released slowly after exposure to collagenase as expected in vivo. These results support gelatin being an effective drug delivery system given gradual release of the cocktail over time. It is important to consider, however, that physiological conditions vary greatly from this in vitro study and that factors including body temperature, relative concentrations of collagenase in the larynx, and physical state of gelatin at the time of injection can all play important roles in the release of cocktail biomolecules. Further efforts to test drug release at longer time points or at different physical states such as in biosphere form is warranted given consistently improved results in nerve regeneration and muscle atrophy attenuation in the G+C treatment groups.

**Discussion**

Though there are several aspects of the neuromuscular junction that are vital in maintaining muscle function, the biomolecules of interest in this paper include agrin, acetylcholine, and neuregulin given their vital role in aggregating and stabilizing acetylcholine receptors. As illustrated in **Figure 1**, agrin is a large proteoglycan that binds to the receptor, Lrp4, leading to subsequent dimerization of Lrp4, activation of MuSK, and phosphorylation of Doc7.^1^ The final step in this pathway is the activation of Rapsyn, which ultimately leads to the invagination and congregation of the acetylcholine receptors (AchR) on the cell membrane of myotubes.^2^ Though acetylcholine is the primary biomolecule in promoting acetylcholine receptor stabilization with prevention of degradation, its function is reinforced with neuregulin, which also plays a major role increasing the number of AchR.^3,4^

Specific examples highlight agrin promoting cardiac regeneration in mice^5^ and reversing significant sarcopenia-like symptoms in NMJ disorders.^6^ Agrin has shown to be a versatile biomolecule useful for wide applications given its effectiveness in promoting acetylcholine receptor stability when used in conjunction with other biomolecules such as neuregulin and laminin.^7-12^ Neuregulin 1 (NRG-1) works synergistically with agrin to increase AChR cluster size through direct interactions between NRG1’s heparin-binding domain and agrin induced ErbB receptor tyrosine kinase activity.^11,12^ In addition to NRG1’s role in upregulating acetylcholine receptor expression and clustering, NRG1 is essential for many areas of neuronogenesis, including axonal myelination and thalamocortical axon guidance.^13,14^ ACh works synergistically with Agrin and NRG1 by activating the AChRs, thereby stabilizing the newly formed motor endplates.^15^

Supplemental References

1. Höbartner A. Development of a heterologous model system for Agrin-Lrp4-MuSK signaling and analysis of muscle-specific kinase endocytosis. Wien, 2015:94 S. : Ill., graph. Darst.
2. Li X, Xu Y, Si JX, Gu F, Ma YY. Role of Agrin in tissue repair and regeneration: From mechanisms to therapeutic opportunities (Review). Int J Mol Med 2024; 54.
3. Li Q, Loeb JA. Neuregulin-heparan-sulfate proteoglycan interactions produce sustained erbB receptor activation required for the induction of acetylcholine receptors in muscle. J Biol Chem. 2001;276:38068–38075. doi: 10.1074/jbc.M104485200.
4. Zhang BG, Quigley AF, Bourke JLet al. Combination of agrin and lamininincrease acetylcholine receptor clustering and enhance functional neuromuscular junction formation In vitro. Dev Neurobiol 2016; 76:551-565.
5. Bassat E, Mutlak YE, Genzelinakh Aet al. The extracellular matrix protein agrin promotes heart regeneration in mice. Nature 2017; 547:179-184.
6. Li X, Xu Y, Si JX, Gu F, Ma YY. Role of Agrin in tissue repair and regeneration: From mechanisms to therapeutic opportunities (Review). Int J Mol Med 2024; 54.
7. Höbartner A. Development of a heterologous model system for Agrin-Lrp4-MuSK signaling and analysis of muscle-specific kinase endocytosis. Wien, 2015:94 S. : Ill., graph. Darst.
8. Yang JF, Cao G, Koirala S, Reddy LV, Ko CP. Schwann cells express active agrin and enhance aggregation of acetylcholine receptors on muscle fibers. J Neurosci 2001; 21:9572-9584.
9. Zhang BG, Quigley AF, Bourke JLet al. Combination of agrin and laminin increase acetylcholine receptor clustering and enhance functional neuromuscular junction formation In vitro. Dev Neurobiol 2016; 76:551-565.
10. Hettwer S, Lin S, Kucsera Set al. Injection of a soluble fragment of neural agrin (NT-1654) considerably improves the muscle pathology caused by the disassembly of the neuromuscular junction. PLoS One 2014; 9:e88739.
11. Ngo ST, Cole RN, Sunn N, Phillips WD, Noakes PG. Neuregulin-1 potentiates agrin-induced acetylcholine receptor clustering through muscle-specific kinase phosphorylation. J Cell Sci 2012; 125:1531-1543.
12. Yang JF, Cao G, Koirala S, Reddy LV, Ko CP. Schwann cells express active agrin and enhance aggregation of acetylcholine receptors on muscle fibers. J Neurosci 2001; 21:9572-9584.
13. Lopez-Bendito G, Cautinat A, Sánchez JA, et al. Tangential neuronal migration controls axon guidance: a role for neuregulin-1 in thalamocortical axon navigation. Cell. 2006;125:127–142. doi: 10.1016/j.cell.2006.01.042.
14. Michailov GV, Sereda MW, Brinkmann BG, et al. Axonal neuregulin-1 regulates myelin sheath thickness. Science. 2004;304:700–703. doi: 10.1126/science.1095862.
15. Cisterna BA, Vargas AA, Puebla Cet al. Active acetylcholine receptors prevent the atrophy of skeletal muscles and favor reinnervation. Nat Commun 2020; 11:1073.
